# Prevalence of rheumatoid arthritis in low– and middle–income countries: A systematic review and analysis

**DOI:** 10.7189/jogh.05.010409

**Published:** 2015-06

**Authors:** Igor Rudan, Simrita Sidhu, Angeliki Papana, Shi–Jiao Meng, Yu Xin–Wei, Wei Wang, Ruth M. Campbell–Page, Alessandro Rhyll Demaio, Harish Nair, Devi Sridhar, Evropi Theodoratou, Ben Dowman, Davies Adeloye, Azeem Majeed, Josip Car, Harry Campbell, Wei Wang, Kit Yee Chan, Global Health Epidemiology Reference Group (GHERG)

**Affiliations:** * Joint first authorship; 1 The University of Edinburgh Medical School, Edinburgh, Scotland, UK; 2 University of Macedonia, Thessaloniki, Greece; 3 School of Public Health, Capital Medical University, Beijing, China; 4 Department of Integrated Early Childhood and Development, Capital Institute of Paediatrics, Beijing, China; 5 The University of Toronto, Toronto, Canada; 6 Copenhagen School of Global Health, University of Copenhagen, Denmark; 7 Harvard Global Equity Initiative, Harvard Medical School, Cambridge, USA; 8 School of Public Health, Imperial College London, London, UK; 9 Lee Kong Chian School of Medicine, Nanyang Technological University, Singapore; 10 Edith Cowan University, Perth, Australia; 11 School of Public Health, Peking University Health Science Centre, Beijing, China

## Abstract

**Background:**

Rheumatoid arthritis (RA) is an autoimmune disorder that affects the small joints of the body. It is one of the leading causes of chronic morbidity in high–income countries, but little is known about the burden of this disease in low– and middle–income countries (LMIC).

**Methods:**

The aim of this study was to estimate the prevalence of RA in six of the World Health Organization's (WHO) regions that harbour LMIC by identifying all relevant studies in those regions. To accomplish this aim various bibliographic databases were searched: PubMed, EMBASE, Global Health, LILACS and the Chinese databases CNKI and WanFang. Studies were selected based on pre–defined inclusion criteria, including a definition of RA based on the 1987 revision of the American College of Rheumatology (ACR) definition.

**Results:**

Meta–estimates of regional RA prevalence rates for countries of low or middle income were 0.40% (95% CI: 0.23–0.57%) for Southeast Asian, 0.37% (95% CI: 0.23–0.51%) for Eastern Mediterranean, 0.62% (95% CI: 0.47–0.77%) for European, 1.25% (95% CI: 0.64–1.86%) for American and 0.42% (95% CI: 0.30–0.53%) for Western Pacific regions. A formal meta–analysis could not be performed for the sub–Saharan African region due to limited data. Male prevalence of RA in LMIC was 0.16% (95% CI: 0.11–0.20%) while the prevalence in women reached 0.75% (95% CI: 0.60–0.90%). This difference between males and females was statistically significant (*P* < 0.0001). The prevalence of RA did not differ significantly between urban and rural settings (*P* = 0.353). These prevalence estimates represent 2.60 (95% CI: 1.85–3.34%) million male sufferers and 12.21 (95% CI: 9.78–14.67%) million female sufferers in LMIC in the year 2000, and 3.16 (95% CI: 2.25–4.05%) million affected males and 14.87 (95% CI: 11.91–17.86%) million affected females in LMIC in the year 2010.

**Conclusion:**

Given that majority of the world’s population resides in LMIC, the number of affected people is substantial, with a projection to increase in the coming years. Therefore, policy makers and health–care providers need to plan to address a significant disease burden both socially and economically.

In recent years there has been a shift in diseases and health related challenges that the world is facing. Non–communicable diseases (NCD) have emerged as the leading cause of death worldwide, accounting for two–thirds of all deaths and deaths are projected to increase in the coming years [1]. Contrary to popular belief, these diseases are not limited to the developed world; they are increasingly prevalent in low– and middle–income countries (LMIC), which are facing the double burden of both communicable and non–communicable diseases [2]. In LMIC, constrained health care facilities, lack of resources and funds at individual and national level lead to limited treatment and support for NCD, which mainly affect the working age population with a negative impact on household incomes and equity. The high burden of NCD poses additional problems for LMIC, creating a vicious cycle by worsening poverty that in turn results in a further rise of NCD [1]. Acknowledgment of the serious implications of the global burden of NCD has led to an international response. The United Nations (UN) High–level meeting on NCD in 2011 addressed these issues and has paved the way for tackling them, by providing guidance on how to strengthen national capacities to address NCD and integrate prevention and control activities across sectors and at all levels of governance and health–care provision in LMIC [1,2].

While there is wide recognition of the four main NCD with a major contribution to the global burden – cardiovascular diseases, cancers, diabetes mellitus and chronic respiratory illnesses [2] – there are a large number of other NCD that cause extensive morbidity, but are neglected as they do not significantly contribute to mortality. One such disease is rheumatoid arthritis (RA), the most common type of inflammatory musculoskeletal disorder [3,4], in which the quality of life has been reported to be lower than in patients suffering from most of the other NCD [5,6]. It is a chronic systematic autoimmune inflammatory disease, characterised by a symmetrical persistent synovitis of the joints of the hands, wrist, feet and knee resulting in tender swelling of joints, pain, limitation in motion and morning stiffness. Its systematic features include fatigue, generalised weakness, loss of weight and low grade fever [7]. As the disease advances, irreversible tissue damage occurs, with destruction of bone and cartilage leading to joint deformity, muscle atrophy, and progression that may involve all joints of the body [8]. For the purpose of clinical trials, RA is diagnosed using the American College of Rheumatology (ACR) criteria, formerly known as the American Rheumatology Association (ARA) criteria [7,9].

The prevalence of RA in the western world is 1–2% [10], and is believed to be 1% worldwide [11]. However, this global estimate is based on a few sporadic studies over different time periods, in a limited number of LMIC. Extrapolation from a few studies is problematic given that there is ample evidence that RA is a variable disease in time and place [11]. Moreover, the burden of NCD has increased over the past decade in LMIC, while it has decreased in high–income countries [11]. RA also has a substantial economic impact, which can be quantified as direct (cost of medication, hospital stay and visits, care–givers and helpers); indirect (loss of productivity from absenteeism or early retirement); and intangible costs that are measured by the impact on quality of life [12,13]. In the United States, the direct cost of RA was approximately US$ 13 500 per affected person per year, and indirect costs could range between US$ 1000 and US$ 33 000 per affected person per year [14]. However, not much is known about costs in the developing world [13].

This paper aims to provide an estimate for the global and regional burden of rheumatoid arthritis by systematically reviewing relevant literature in both English databases and those in other languages; to study the variation in the prevalence of rheumatoid arthritis by gender, region and setting (urban/rural); and to discuss the significance of these prevalence estimates and their implications for public health policy.

## METHODS

### Definition of population under study and literature search

The World Bank database was referenced to compile a list of all the LMIC in the world [15]. Thereafter, all LMIC were grouped into regions in accordance to the World Health Organization (WHO) regions [16]. WHO divides the world into six regions; Southeast Asian Region (SEAR), Eastern Mediterranean Region (EMR), Western Pacific Region (WPR), Europe (EUR), The Americas (AMR) and Sub–Saharan Africa (AFR) [17–19]. A systematic literature search was conducted separately for each region to find population based studies that documented the prevalence of RA. Medline (1946 – July week 1, 2013), EMBASE (1976– 2013 week 26) and Global Health (GH) (1973 – 2013 week 26) were searched using the OVID search engine (search terms available in **Online Supplementary Document(Online Supplementary Document)**). Both Medical Subject Headings (MeSH terms) and keywords were used in OVID. Other online databases such as PubMed, Web of Knowledge (WoK) and databases selective to regions, such as LILACS for Latin America, CNKI and Wan Fang for China, and IndMed for India, were also thoroughly searched. PubMed was searched for all regions, as it proved to be broader and more sensitive in picking up studies. Grey literature was also searched for all low–middle income countries using SIGLE (OpenGrey), Google Scholar and Global Health library. The search of grey literature resulted in 10 and 149 studies, respectively, none of which were relevant to this analysis.

### Inclusion and exclusion criteria for study selection

After the initial screen, inclusion and exclusion criteria were applied to retain only the studies that were free of any apparent bias. We included studies conducted in LMIC from all WHO regions that were population based or community based, studies conducted after 1987 that used ARA/ACR diagnostic criteria (see **Table 1**), focused on adult populations (typically 15+ or 18+ years, with exclusion of juvenile forms in the former studies) and reported the prevalence rate of RA. We excluded review articles with secondary data only (with the exception of sub–Saharan Africa, where the amount of data was particularly scarce), hospital–based studies (for lack of representativeness of the general population), studies conducted prior to or during 1987 (for inconsistent case definition), studies on other types of arthritis in adults, studies on juvenile forms of arthritis and studies that used other diagnostic criteria to measure the prevalence of RA in the population.

**Table 1:** The criteria of the American College of Rheumatology (ACR) established in 1987 to assist clinical diagnosis of rheumatoid arthritis*

1. Morning stiffness	Morning stiffness in and around the joints, lasting at least 1 hour before maximal improvement
2. Arthritis of 3 or more joints	At least 3 joint areas simultaneously have had soft tissue swelling or fluid (not bony overgrowth alone) observed by a physician. The 14 possible areas are right or left PIP, MCP, wrist, elbow, knee, ankle, and MTP joints
3. Arthritis of hand joints	At least 1 area swollen (as defined above) in a wrist, MCP, or PIP joint
4. Symmetric arthritis	Simultaneous involvement of the same joint areas (as defined in 2) on both sides of the body (bilateral involvement of PIPs, MCPs, or MTPs is acceptable without absolute symmetry
5. Rheumatoid nodules	Subcutaneous nodules, over bony prominences, or extensor surfaces, or in juxta–articular regions, observed by a physician
6. Serum rheumatoid factor	Demonstration of abnormal amounts of serum rheumatoid factor by any method for which the result has been positive in <5% of normal control subjects
7. Radiographic changes	Radiographic changes typical of rheumatoid arthritis on postero–anterior hand and wrist radiographs, which must include erosions or unequivocal bony decalcification localized in or most marked adjacent to the involved joints (osteoarthritis changes alone do not qualify

PIP – proximal interphalangeal; MCP – metacarpophalangeal; MTP – metatarsophalangeal

*Four out of the seven criteria need to be met in order to establish the diagnosis of RA, with criteria 1–4 required to be present for at least 6 weeks. Patients with two clinical diagnoses are not excluded [9].

We retained studies that clearly presented the method of diagnosing RA, beginning with how the sample population was recruited and evaluated, along with the criteria used for diagnosis of RA. We expected that trained personnel or specialists be involved in the field work, and we excluded the studies where self–reporting was the primary method of case ascertainment. Specialists (doctors, rheumatologists) needed to be involved in the next step of confirmation. Any study where there was no direct contact between the assessors and sample population, such as telephone surveys, were excluded. There is a high probability of misclassification and oversight of cases by untrained or inadequately trained personnel, or through indirect contact.

**Figure 1:**
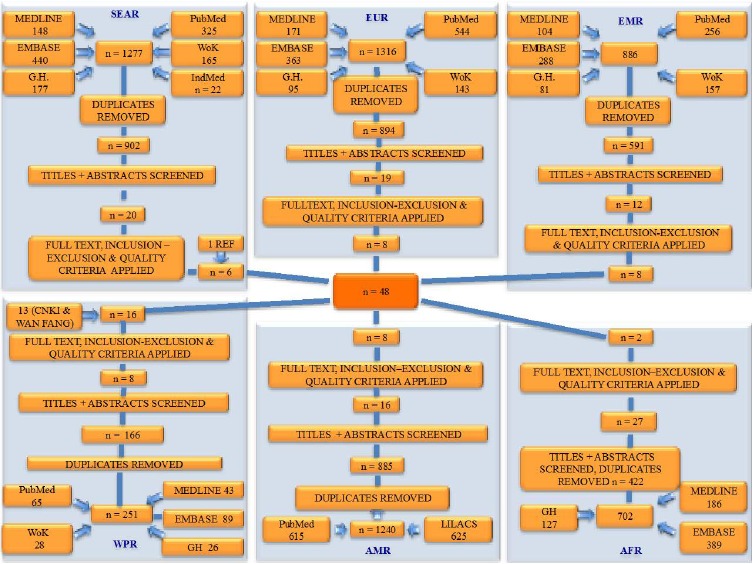
Flowchart presenting the literature search and the process of study selection (WoK = Web of Knowledge; G.H. = global health).

**Figure 1** summarises the process of study selection for all six WHO regions. First, duplicates were excluded and titles and abstracts of the retained papers were evaluated for relevant studies. Full texts of selected studies were analysed and inclusion and exclusion criteria were applied. Data from all relevant studies was extracted into an Excel spreadsheet, where sample size (age, sex–specific, mean age), methodology, criteria used for diagnosing RA, study location (urban or rural) and prevalence rate were documented for each study.

### Adjustment of prevalence rates

Once the final set of studies was retained (**Figure 1**), crude prevalence rates, sex–specific prevalence rates, urban and rural prevalence rates and male–to–female ratio of RA cases were adjusted to the same measurement unit and expressed as a percentage. Data extracted from each study is shown in the **Online Supplementary Document(Online Supplementary Document)**. Checks for internal consistency of the data were made and possible significant correlations between prevalence rate and the sample size, year of publication of the study, sex and residency were made.

#### Statistical analyses

All statistical analyses are shown in the **Online Supplementary Document(Online Supplementary Document)**. We first tested the distribution of the reported prevalence of RA across all identified studies for normality using the one–sample Kolmogorov-Smirnov test. We concluded that the results did not indicate normal distribution, presumably because of substantial heterogeneity in the included studies (Z = 1.831, *P* = 0.002). We then performed a meta–analysis of all identified studies in all LMIC using the DerSimonian–Laird method, to determine the “LMIC” prevalence rate (**Online Supplementary Document(Online Supplementary Document)**).

**Figure 2:**
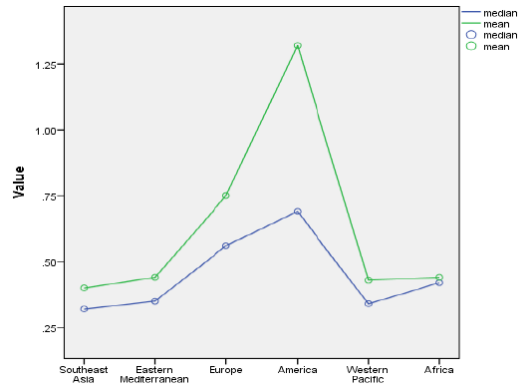
The relationship between mean and median prevalence of rheumatoid arthritis in low and middle–income countries in six WHO regions of the world.

We then displayed mean and median prevalences in each of the six WHO regions (**Figure 2**). The Kruskal-Wallis one–way analysis of variance by ranks examined whether samples originated from the same distribution. After this, we conducted a series of region–specific meta–analyses to estimate regional prevalence and confidence intervals, using the DerSimonian–Laird method (**Online Supplementary Document(Online Supplementary Document)**).

An important possible confounding effect was differences in the mean age of the sample between the studies. This is because, although all samples were defined as “adult population” (usually 15 years of age or older), the relative contribution of elderly population varied in different countries because of difference in sampling strategies and overall life expectancy. We explored the association between the prevalence of RA and the mean age using linear correlation coefficients (Pearson, Kendall’s tau, and Spearman), and also the generalised dependence measure mutual information to exclude the potential effect of age distribution on generalisability of the results (**Online Supplementary Document(Online Supplementary Document)**).

Association between gender and prevalence of RA, where a considerable difference between sexes was expected, was explored using the paired samples t–test. We then conducted a gender–specific meta–analysis to estimate prevalence and confidence intervals in men and women, using the DerSimonian–Laird method. In addition, box–and–whiskers plots of regional prevalence by gender were also presented (**Online Supplementary Document(Online Supplementary Document)**). We also examined the difference in prevalence of RA between urban and rural populations. Since the RA prevalence is not normally distributed, we performed the non–parametric Mann-Whitney U test to test the null–hypothesis.

## RESULTS

In our study, the majority of studies were from mainland China, with additional studies from Taiwan and Hong Kong. Mexico, Turkey, Iran, India, Pakistan, Philippines and Russia were also represented through multiple studies. The median year of publication was 2004, making the estimate useful for application to both the world population in 2000 and 2010. Twenty–one studies used the ARA criteria, and all the remaining studies used the 1987 revised ACR criteria. Some studies had multiple cohorts. Each cohort was recognised separately during analysis, so that the final 48 studies resulted in 60 cohorts. In case of the African region, only two studies were found from the entire region that fulfilled the criteria for inclusion. One of the studies had a very small sample size and did not find a single case of RA, so it was excluded as uninformative. The other study had a crude prevalence rate of 1%, but we felt that it would be inadequate to base an entire regions' prevalence rate on a single study. Therefore, we decided to discard both of those studies and replace them by Bowman’s systematic analysis in 2012 [20]. Bowman included all the studies ever conducted in Africa in his estimate, irrespective of the year of study, and the prevalence rate from his study was then applied to the population statistics of the region in 2000 and 2010.

**Figure 3:**
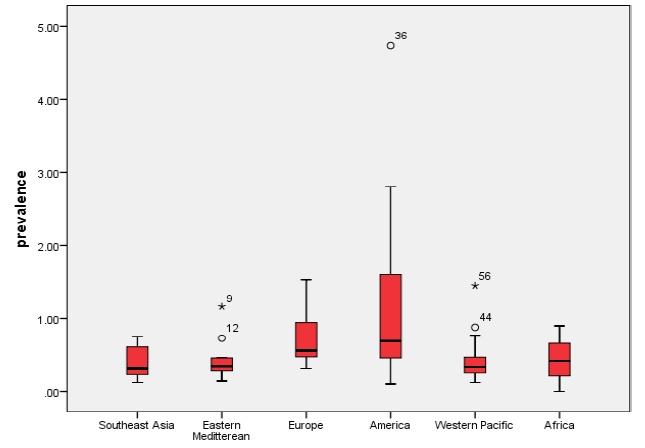
Regional median, minimum and maximum observed value and inter–quartile range for the prevalence of rheumatoid arthritis in low and middle–income countries in six WHO regions of the world.

When all studies were analysed in one large meta–analysis, irrespective of their heterogeneity, this resulted in an “LMIC” estimate for the prevalence of RA of 0.53% (95% CI: 0.45–0.61%). Analysis of heterogeneity confirmed that the data were highly heterogeneous (I^2^ = 96%) (**Online Supplementary Document(Online Supplementary Document)**). We then studied the mean and median prevalence in each of the six WHO regions (**Figure 2**) and presented box–and–whiskers plot of the results from studies in each region (**Figure 3**). The Kruskal-Wallis one–way analysis of variance by ranks test showed that the prevalence in at least one of the WHO regions was statistically different from the others (*P* = 0.029).

A series of region–specific meta–analyses were conducted to estimate regional prevalence of RA. The meta–analysis estimates of regional RA prevalence rates were 0.40% (95% CI: 0.23–0.57%) for Southeast Asia, 0.37% (95% CI: 0.23–0.51%) for Eastern Mediterranean, 0.62% (95% CI: 0.47–0.77%) for European LMIC countries, 1.25% (95% CI: 0.64–1.86%) for American LMIC countries and 0.42% (95% CI: 0.30–0.53%) for Western Pacific LMIC countries, respectively. This analysis could not be performed for Africa due to limited data. The data sets were heterogeneous in all the regions (I^2^ varied from 74.2% to 97.3%).

We then studied whether the mean age of the sample contributed to the observed prevalence rates. Linear correlation coefficients (Pearson, Kendall’s tau, and Spearman) and the generalised dependence measure mutual information did not show an effect of mean age of the sample on the reported prevalence of RA across the studies (*P* = 0.0599; *P* > 0.05), implying that the differences in age structure of samples in different studies were not the main determinant of the observed heterogeneity.

**Figure 4:**
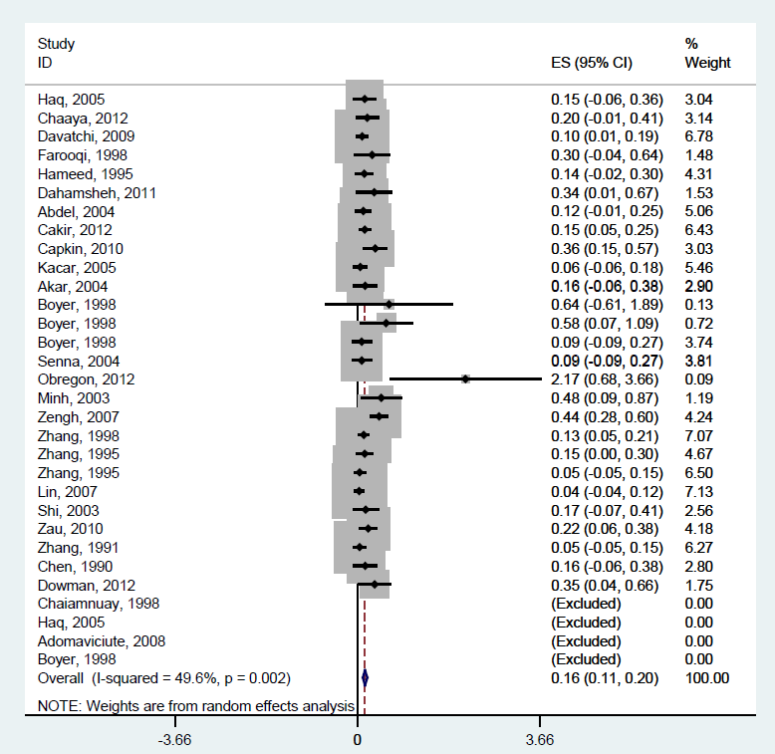
Meta–analysis of rheumatoid arthritis prevalence in men, based on all available information from low and middle–income countries in six WHO regions of the world.

**Figure 5:**
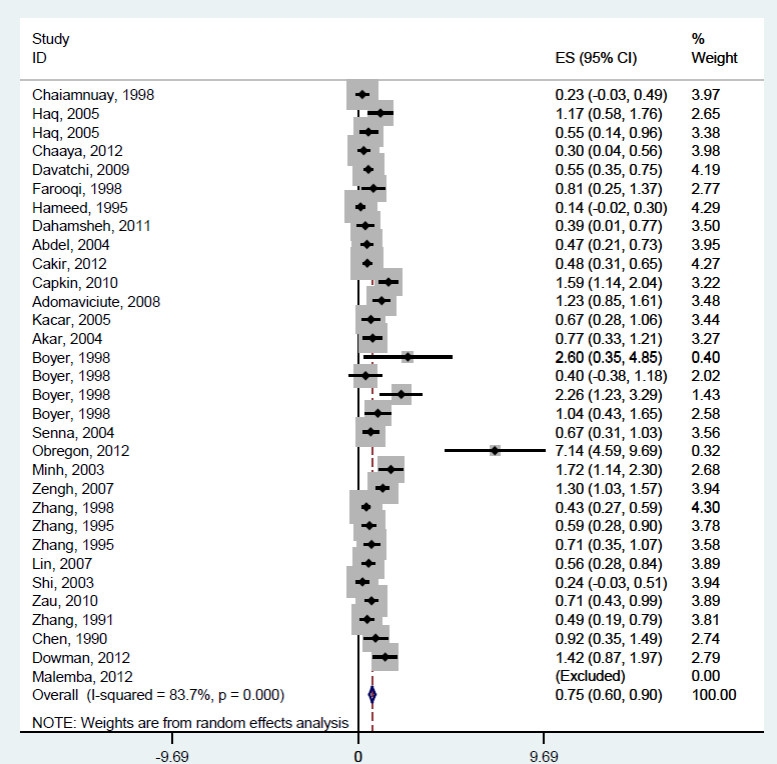
Meta–analysis of rheumatoid arthritis prevalence in women, based on all available information from low and middle–income countries in six WHO regions of the world.

An investigation into differences in prevalence by gender using paired samples t–test indicated that the male and female RA prevalence differed significantly (*P* < 0.0001), which was expected. We therefore conducted a separate meta–analysis of the RA prevalence in LMIC countries for men and women. Male prevalence was 0.16% (95% CI: 0.11–0.20%) (**Figure 4**) while the prevalence in women was five times higher, amounting to 0.75% (95% CI: 0.60–0.90%) (**Figure 5**). Data seemed to be less heterogeneous for men (I^2^ = 49.6%) than for women (I^2^ = 83.7%).

**Figure 6:**
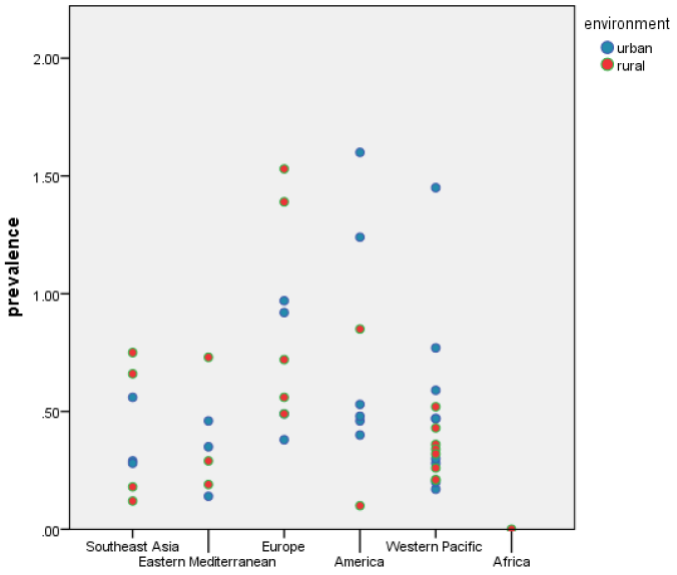
A scatterplot of observed prevalence rates of rheumatoid arthritis in six WHO regions based on urban or rural residency of the examinees. No statistical differences were noted (see **Online Supplementary Document(Online Supplementary Document)** for further detail).

We also examined the difference in prevalence of RA between urban and rural populations, wherever information was available to allow for comparison. Since we established that the RA prevalence was not normally distributed, we performed the non–parametric Mann-Whitney U test to test the null hypothesis. The significance of the test was *P* = 0.353, indicating that the prevalence in the urban and rural settings do not differ significantly (**Figure 6**).

After all the previous analyses, a strategy was needed for estimating the number of persons living with RA in LMIC in the years 2000 and 2010. Possible approaches were: (i) to apply the meta–analysis of the crude prevalence from all identified studies to the total number of persons 15 years or older in LMIC; (ii) to use the estimate of prevalence for males and for females that resulted from the meta–analyses of all studies that reported the rates separately by gender; then, to apply those two estimates of prevalence to male and female populations in LMIC; (iii) to use regional medians or the estimates based on regional meta–analysis and apply them to the regional populations aged 15 years or older; (iv) finally, to use sex–specific regional estimates and apply it to male and female population aged 15 years or older in respective regions.

Given the quantity and quality of the information that was obtained through this systematic review, the most appropriate (and robust) approach was to use gender–specific estimates of prevalence for the whole LMIC region and apply them to male and female populations in LMIC. There are a number of reasons why other approaches were not preferred and we will list them here. Although the quantity of information was the largest for the approach (i) above, there is uncertainty in some studies over the composition of sample by gender, and whether it is representative of the underlying population. Given that gender is an extremely important determinant of prevalence, the approach (i) would suffer from a possible confounding effect of the gender composition of the sample. The strength of approach (iii) was that it could account for regional variation. However, the number of studies typically available for different regions was simply too small to be sure whether the observed differences between regions were real, or just stochastic. The same applies to approach (iv). Therefore, approach (ii) was used, because it accounted for the most important confounding variable – gender – and because it provided a lot of information for meta–analysis in each gender. This allowed an assumption that the rates considered representative for all males and all females in LMIC were more likely to be accurate than region–specific rates. Moreover, the observed heterogeneity of the underlying data was the lowest in gender–specific meta–analysis across LMIC.

This gives an estimate of male prevalence of 0.156% (95% CI: 0.11–0.20%) (**Figure 4**) that needs to be applied to the male population aged 15 years or more in LMIC in 2000 and 2010. In females, the prevalence of 0.747% (95% CI: 0.60–0.90%) is used (**Figure 5**). The UN Population Division's estimates for the number of males aged 15 years or older in LMIC in the year 2000 is 1.667 billion, and in the year 2010 it is 2.206 billion [17]. For women, the corresponding figures are 1.634 billion for the year 2000 and 1.991 billion in 2010 [17]. This translates into 2.60 (95% CI: 1.85–3.34%) million male sufferers and 12.21 (95% CI: 9.78–14.67%) million female sufferers in the year 2000, and 3.16 (95% CI: 2.25–4.05%) million affected males and 14.87 (95% CI: 11.91–17.86%) million affected females in the year 2010 in the countries of low and middle income.

## DISCUSSION

We presented a robust estimate of the number of individuals suffering from RA in low and middle income countries in 2000 and 2010. There have already been several attempts to estimate the prevalence of RA at the global, regional and national level and also in LMIC [10,11,14,18,19]. In comparison to previous estimates that presented both higher and lower estimates than our study, our estimate is based primarily on a substantial amount of evidence from LMIC on sex–specific prevalence. We demonstrated that gender is a principal determinant of RA in LMIC and that age distribution of the population and being an urban dweller do not contribute significantly to disease development. This is different from some other diseases, such as dementia and cancer, where age seems to be the main determinant, or schizophrenia, where being an urban dweller and family history seem to be the main driver of the disease occurrence [1,2]. Therefore, we believe that our strategy for deriving the estimate was more appropriate than used in some previous studies. Moreover, we provide the full data set used to develop the estimates in **Online Supplementary Document(Online Supplementary Document)** and all our methods are transparent and replicable by other groups.

Still, there are limitations in all estimates of the current global burden of RA. The criteria of defining the disease have changed over time and the estimates that don't take this into account will be internally inconsistent. Moreover, a mixture of studies, both hospital and population based studies, could be considered, although the former will present the more severe end of disease spectrum and bias the results. A major strength of our study is that it made use of all literature available in all languages, including two major Chinese databases and the database with grey literature. We therefore believe that we are presenting the most advanced estimate of RA burden to date. However, limitations are still large: there are very few data points (particularly in Africa) and most LMIC countries do not have a single published epidemiological study. Moreover, most of the studies used for this estimate are quite small and they are unlikely to be nationally representative. Also, this study uses a wide range of years to provide estimates for 2000 and 2010, which is a limitation given that prevalence may be changing over time and that the time trend reported here arises from demographic changes, rather than our understanding of the epidemiological situation.

Our paper also aimed to explore whether other major covariates, besides gender, affect the frequency of the occurrence of RA. We were unable to demonstrate significant effects for either urban / rural living or age structure of the study sample. Comparing all urban, rural and mixed studies amongst each other, we were unable to demonstrate significant differences between prevalence rates in urban or rural areas. This is contrary to some previous reports that suggested that the prevalence might be higher in urban areas [21]. Moreover, previous reports suggested that the prevalence rate in LMIC is lower than in the developed countries [22], which our study seems to generally support.

One of the major strengths of our study is that it involved a systematic search of ten large databases, resulting in the identification of 10 599 studies initially and 48 studies selected for inclusion. Native speakers translated studies in a language other than English, specifically Chinese and Spanish. This has greatly increased the pool of studies available for analysis, as it led to the inclusion of studies otherwise excluded due to language barriers. All of the studies used the same definition to identify RA in patients: the 1987 revised ACR criteria (previously known as ARA criteria). This enabled comparison and convergence of studies towards a single plausible estimate. Besides three studies, in which we adjusted the estimate, all others determined the prevalence rate using the same age cut–off (15 years or older), again leading to comparable estimates underlying each regional and the overall prevalence rate.

Although the best quality of epidemiological work on RA in LMIC has been conducted by WHO–ILAR–COPCORD [23], this program covers a limited number of countries and this current review includes a larger number of studies conducted elsewhere, by different researchers, leading to wider coverage. Still, nearly all of the retained studies closely followed the three–step methodology set by WHO–ILAR–COPCORD and used similar questionnaires, thereby decreasing the methodological variability in the studies [23]. The questionnaires were translated in local languages and tested before the start of the studies in almost all cases.

Nevertheless, variation remained even within the selected studies that share the same three–step methodology. The assessors at each stage were different between the studies. In some studies, trained nurses administered the questionnaire, while in others this was done by trained volunteers and students. At the second and third stage most studies involved rheumatologists, but a handful of studies had general medical doctors or internists evaluate the potential cases. Moreover, among the studies conducted by COPCORD, there was a slight regional variation in the questionnaire used, given that it was modified over time, decreasing the comparability of studies. Although the number of participants at each step is given, the reason of non–participation is not stated in all studies. This may have led to non–respondent bias. Although this problem cannot be easily controlled, it still needs to be acknowledged, as there may be a difference in characteristics of those who participate and those who do not.

Research in developed countries does not seem to suggest a growing trend in the prevalence of the disease. However, the total number of cases grew considerably between 2000 and 2010 because the population of LMIC older than 15 years has grown in this period [24]. Even a slight increase in the prevalence rate, eg, an additional prevalence of 0.1%, would translate to an increment of 4 million affected persons. Given the fact that RA affects the working age population and most of the employment in these countries is still for manual labour, it greatly decreases the productivity of countries as a whole [25]. Additionally, the high costs of treatment, borne by individuals themselves in the most LMIC, counters other efforts to decrease poverty and improve living standards.

As RA is an important condition with significant morbidity and economic impact, it should be at the forefront in health care policy. Despite this, RA as part of a larger group of NCD receives less than 3% of annual development assistance for health to low and middle income countries. The neglect of NCD on the global stage can be explained not only by the gaps in estimates on burden of disease but also from a lack of strategic communication about the urgency of the problem [26].

In this paper, estimates of RA morbidity aim to take a first step in raising awareness of policy makers and health care workers, as previously they have had to rely on the evidence that was generated mainly in the developed world. The lack of specialists to diagnose and treat this condition should also be addressed. In certain African nations there is only one rheumatologist for the entire population [21]. Therefore, an increase in the number of specialists in this area is urgently needed in LMIC [27]. As this takes time, the existing doctors in the community should be offered specific education on RA, including newer treatment regimens and the management of the associated comorbid conditions. As most of the population resides in rural areas, incorporating identification and treatment of the disease in community health care system is crucial to reach all those in need of diagnosis and treatment. Funding should be targeted at increasing efficacy of treatments in LMIC. Efforts should be made to increase the availability of treatment – both anti–inflammatories and the newer biological agents that have proven to be greatly beneficial – at affordable costs. The newer biological agents are very expensive and it is unlikely that many LMIC could afford to supply them [25]. Moreover, RA is associated with an increased risk of other diseases (such as cardiovascular diseases) and the management of these comorbid conditions is also important. All doctors should be made aware of treatment protocols already in use by high–income countries with emphasis on early treatment, to slow disease progression and elimination of pain. This should lead to improvements in quality of life and decrease the occurrence of co–morbid conditions, such as depression. The set–up of supportive treatment, such as physiotherapy, should be encouraged [11–18].

Infrastructure for research in areas where it is currently unavailable should be set up. Allegiance with international agencies already working towards generating information, such as WHO–ILAR [23], should be undertaken and their efforts should be supported. In Africa, the African League of Associations of Rheumatology (AFLAR) already exists, but little has been done in terms of research and surveillance of rheumatologic diseases [20]. Such associations should be supported, encouraged and pressurised by governments to carry on more work in this area. The WHO–ILAR–COPCORD program was developed to identify all types of musculoskeletal disorders, and not specifically designed for rheumatoid arthritis nor as an epidemiological study program [23]. Therefore, further research should be specifically orientated towards rheumatoid arthritis, with greater attention on the methodology. The lack of information from more than 100 LMIC countries should be addressed and gaps filled. Studies should also include age groups of those with the disorder, thereby providing more information on who bears the greatest burden and allowing age–standardised comparison. Simultaneously, information about risk factors should be obtained and incorporated in study designs.

The estimates presented in this paper provide a building block for future epidemiological studies by suggesting the way forward in disease assessment in LMIC context and the methodology that could be deployed. It could also be used to create awareness among health–care workers and education of people about the disease and encourage health–seeking behaviour for provision of available treatment, which can decrease the burden associated with disability and bring about a decrease in morbidity. It is also noteworthy to point out the reoccurring theme of lack of data from the poorest countries. Policy makers from these countries should show more dedication and step up their efforts towards research in the health care sector, as generating information about the burden of disease is the first step in decreasing its prevalence.
