# Supplementary material for: Prevalence of rheumatoid arthritis in low– and middle–income countries: A systematic review and analysis
Source: J Glob Health. 2015 Apr 24;5(1):010409. doi: 10.7189/jogh.05.010409 (PMC4416333; doi:10.7189/jogh.05.010409)
Supplement: Online Supplementary Document [file jogh-05-010409-s001.pdf]

Online Supplementary Document

Rudan, Sidhu et al. Urbanisation and prevalence of rheumatoid arthritis in low and middle income countries: A systematic review and analysis

J Glob Health 2015;5:010409

CONTENTS

1. Rheumatoid arthritis prevalence in low and middle income countries (LMIC)

2. Estimation of global RA prevalence based on meta-analysis

3. Estimation of regional RA prevalence based on means and medians

4. Estimation of regional RA prevalence based on meta-analysis

5. Association between mean age of the sample and RA prevalence

6. Association between gender and RA prevalence

7. Global RA prevalence for urban / rural environment

8. The full information extracted from the identified studies and used in a systematic analyses

1. Rheumatoid arthritis prevalence in low and middle-income countries (LMIC)

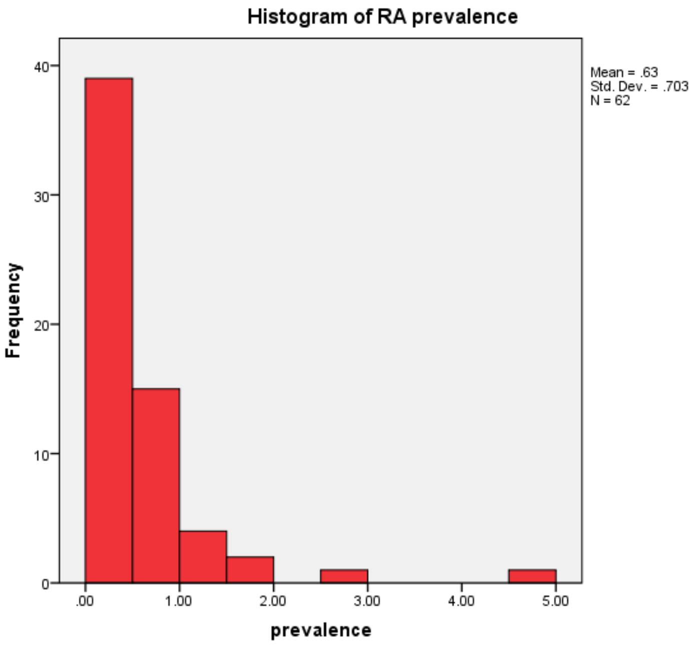

The RA prevalence is not normally distributed (p=0.002).

<u>1.1. One-Sample Kolmogorov-Smirnov Test</u>

		prevalence
N		62
Normal Parameters[a,b]	Mean	.6289
	Std. Deviation	.70348
Most Extreme Differences	Absolute	.233
	Positive	.233
	Negative	-.210
Kolmogorov-Smirnov Z		1.831
Asymp. Sig. (2-tailed)		.002

a. Test distribution is Normal.

b. Calculated from data.

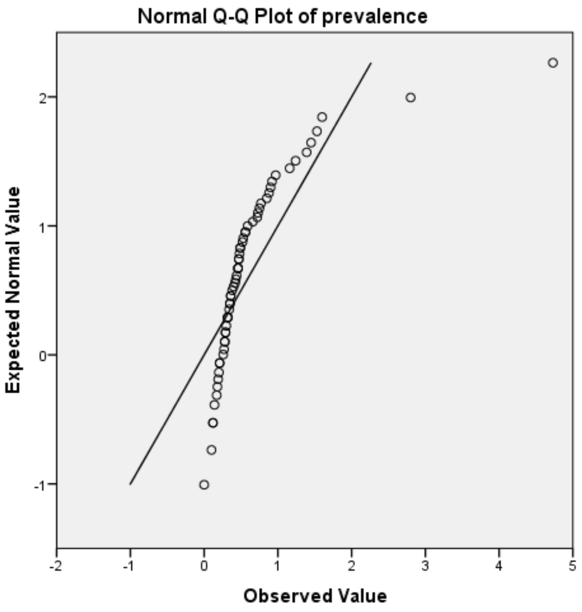

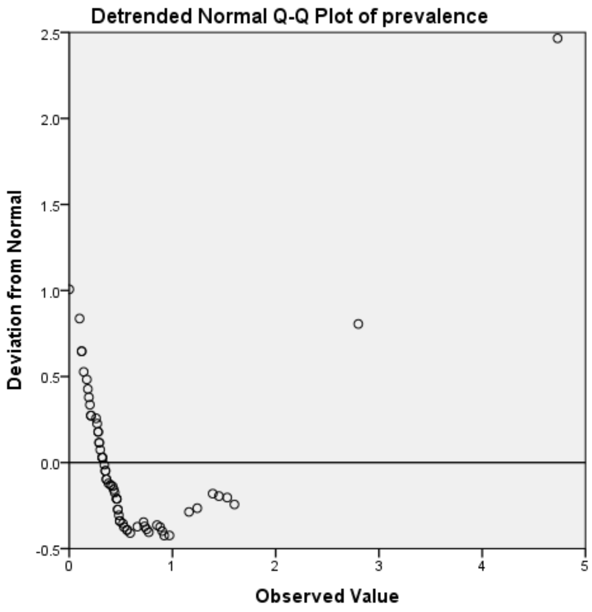

2. Estimation of global RA prevalence based on meta-analysis

The meta-analysis estimate of the global RA prevalence is 0.532 with 95% CI (0.452, 0.612) (DerSimonian-Laird method). Data are highly heterogeneous (I^2 = 96%). The meta-analysis results are displayed graphically with a forest plot (main text, **Figure 2,** and below).

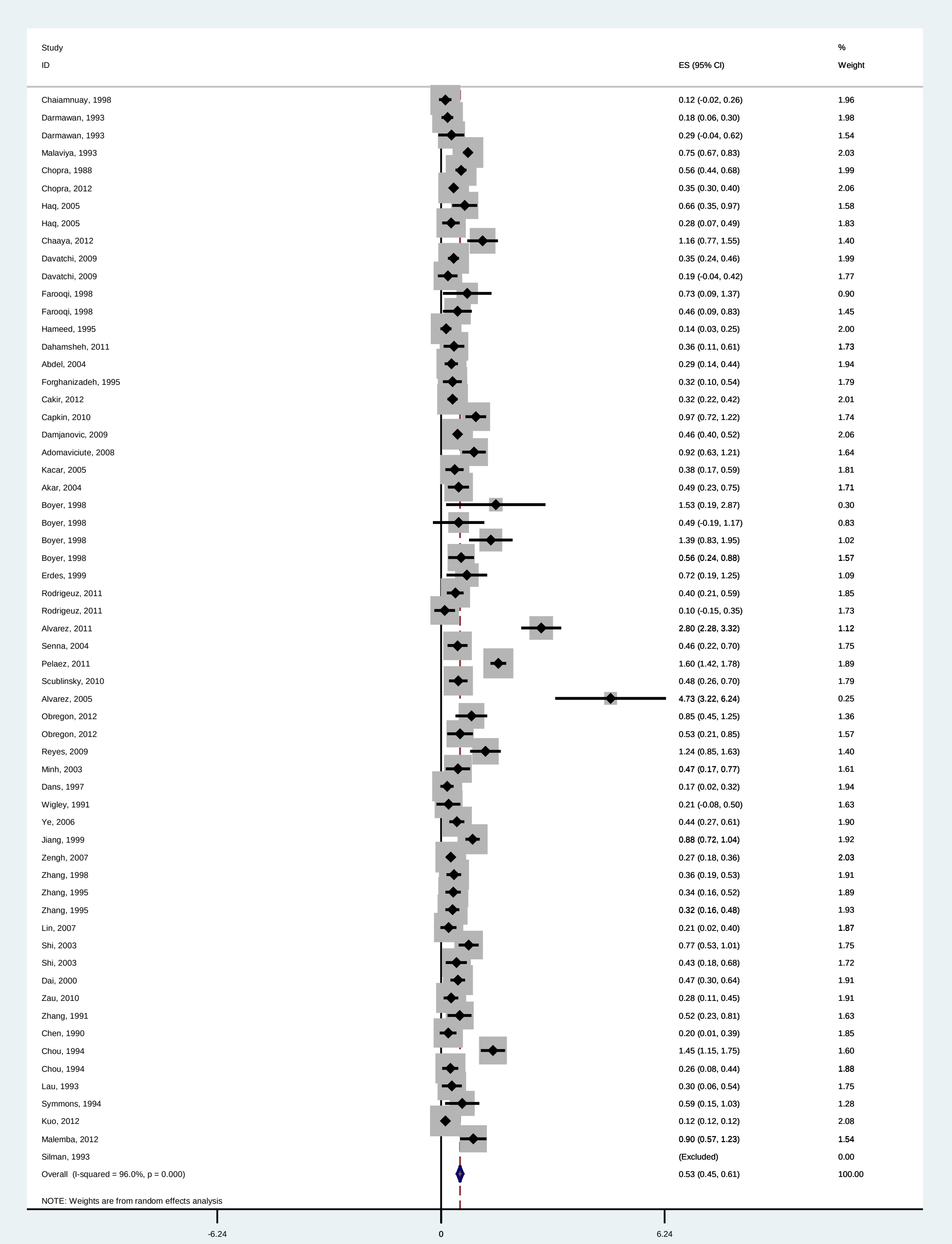


```
Heterogeneity chi-squared = 1489.80 (d.f. = 59) p = 0.000
I-squared (variation in ES attributable to heterogeneity) =  96.0%
Estimate of between-study variance Tau-squared =  0.0804

Test of ES=0 : z=  13.01 p = 0.000
```

3. Estimation of regional RA prevalence based on means and medians

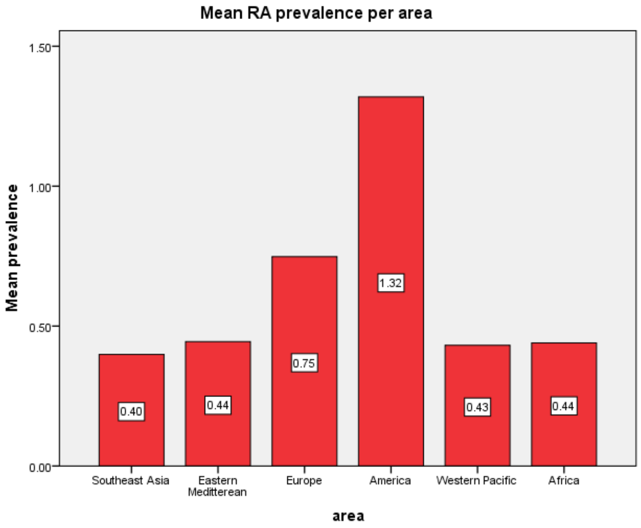

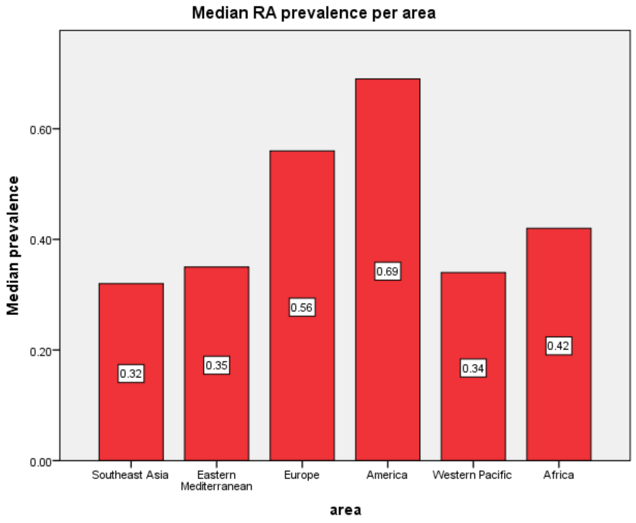

3.1. Kruskal-Wallis test

The Kruskal–Wallis one-way analysis of variance by ranks examines whether samples originate from the same distribution. Since the significance of the test is p=0.029, at least one of the samples is statistically different from the others.

Ranks

	area	N	Mean Rank
prevalence	Southeast Asia	8	25.06
	Eastern Mediterranean	9	25.94
	Europe	11	42.59
	America	10	43.00
	Western Pacific	21	25.64
	Africa	3	27.33
	Total	62	

Test Statistics[a,b]

	prevalence
Chi-Square	12.471
df	5
Asymp. Sig.	.029

a. Kruskal Wallis Test

b. Grouping Variable: area

Since the RA prevalence is not normally distributed, we performed non-parametric Mann-Whitney U test to test the null hypothesis that the populations (considering pairs of areas) are the same. The regional RA prevalence rates that differed significantly were Southeast Asia - Europe (p=0.045),

Eastern Mediterranean – Europe (p=0.031), Eastern Mediterranean – America (p=0.035), Europe - Western Pacific (p=0.006) and America - Western Pacific (p= 0.017).

4. Estimation of regional RA prevalence based on meta-analysis

The meta-analysis estimates of regional RA prevalence rates were 0.397 (95% CI: 0.230-0.565) for Southeast Asia, 0.368 (0.232-0.505) for Eastern Mediterranean, 0.619 (0.469-0.770) for Europe, 1.253 (0.644-1.863) for America, 0.415 (0.304-0.526) for Western Pacific, respectively. No results can be extracted for Africa due to the limited data. Data are heterogeneous at all cases (I^2 varies from 74.2% to 97.3%).

4.1. Meta-analysis results for region Southeast Asia (SEAR):

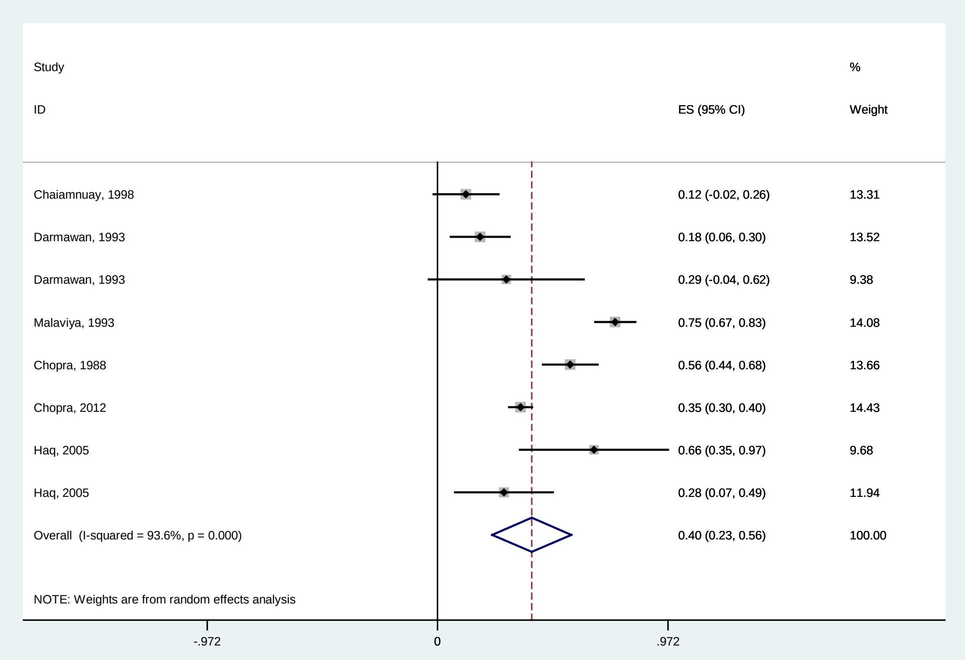

4.2. Meta-analysis results for region Eastern Mediterranean (EMR):

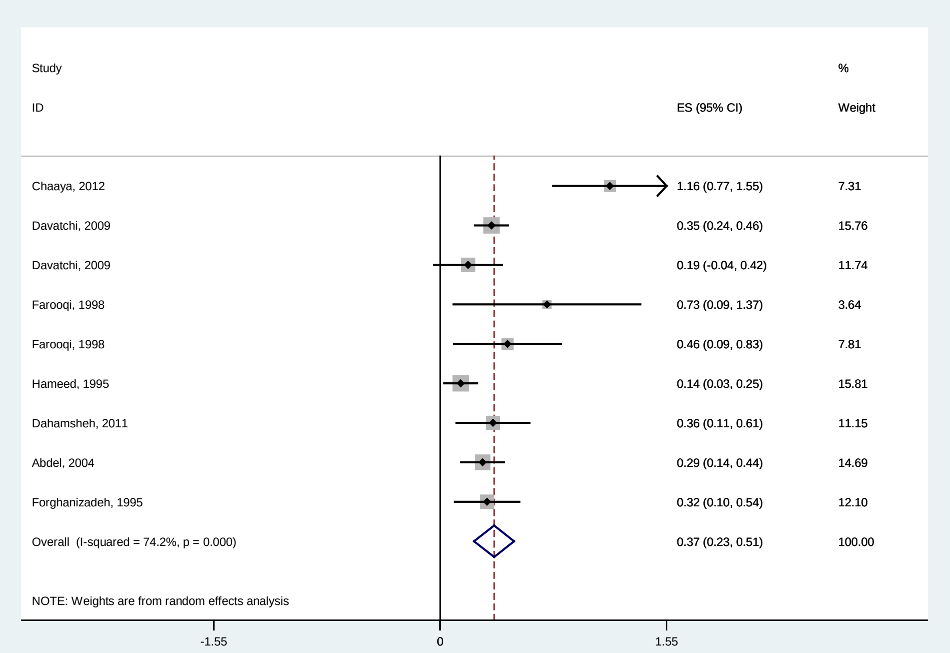

4.3. Meta-analysis results for region Europe:

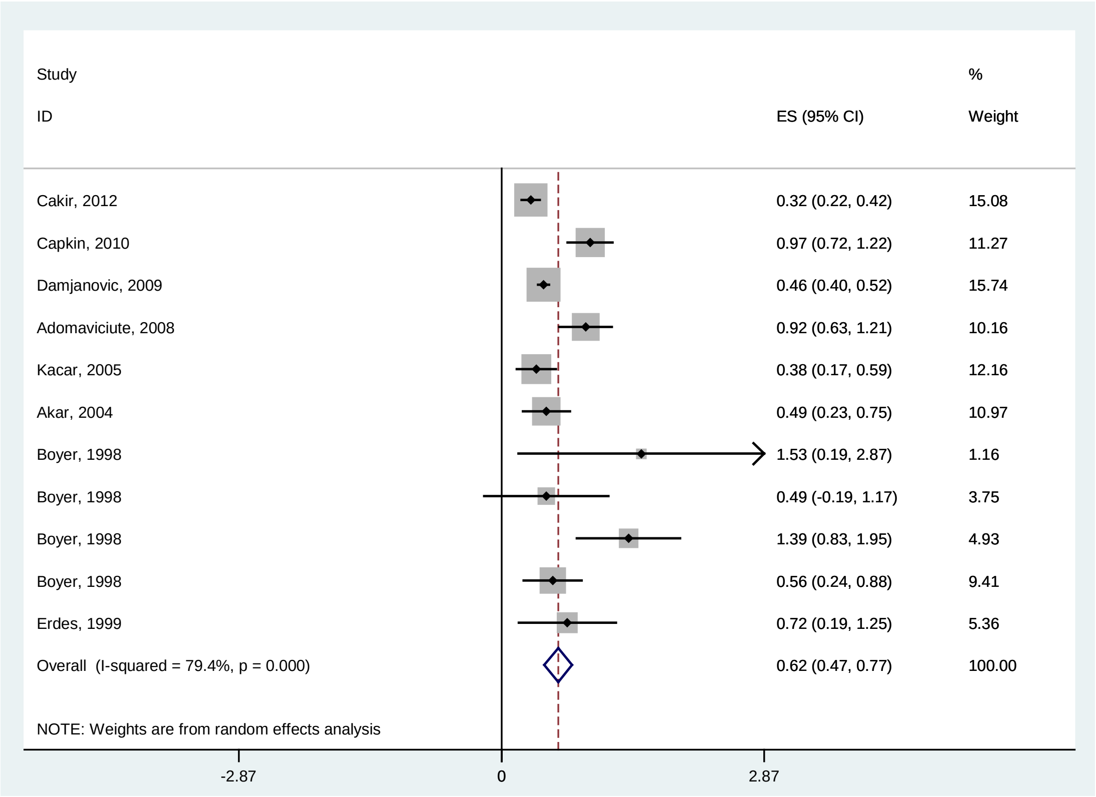

4.4. Meta-analysis results for region America:

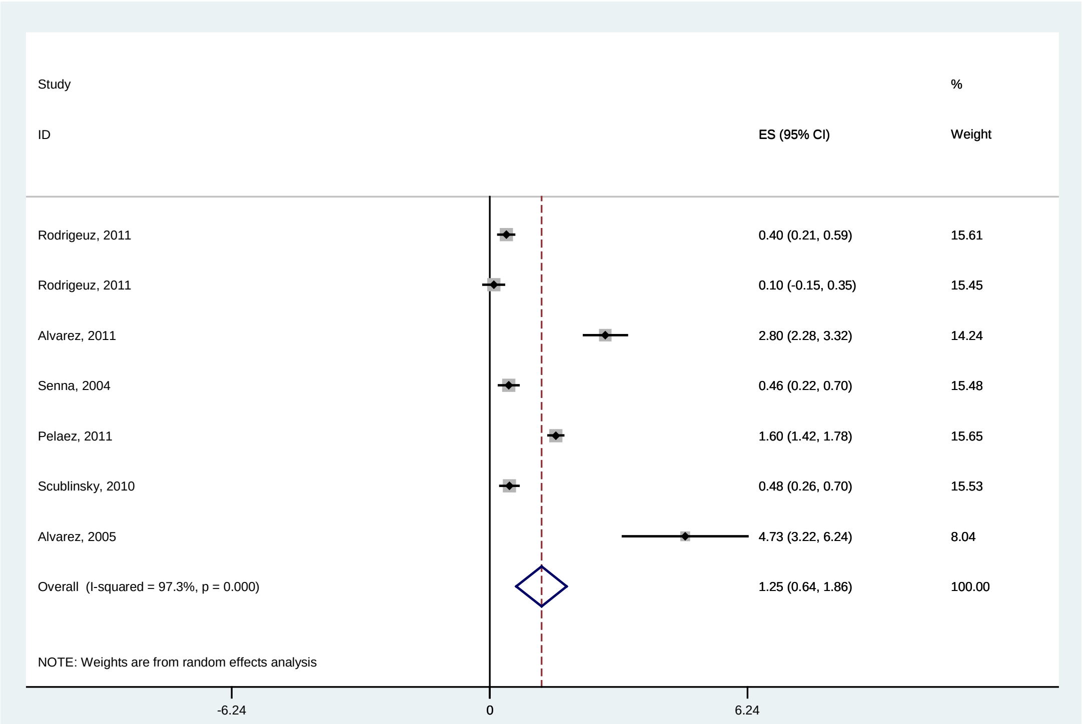

<u>4.5. Meta-analysis results for region Western Pacific:</u>

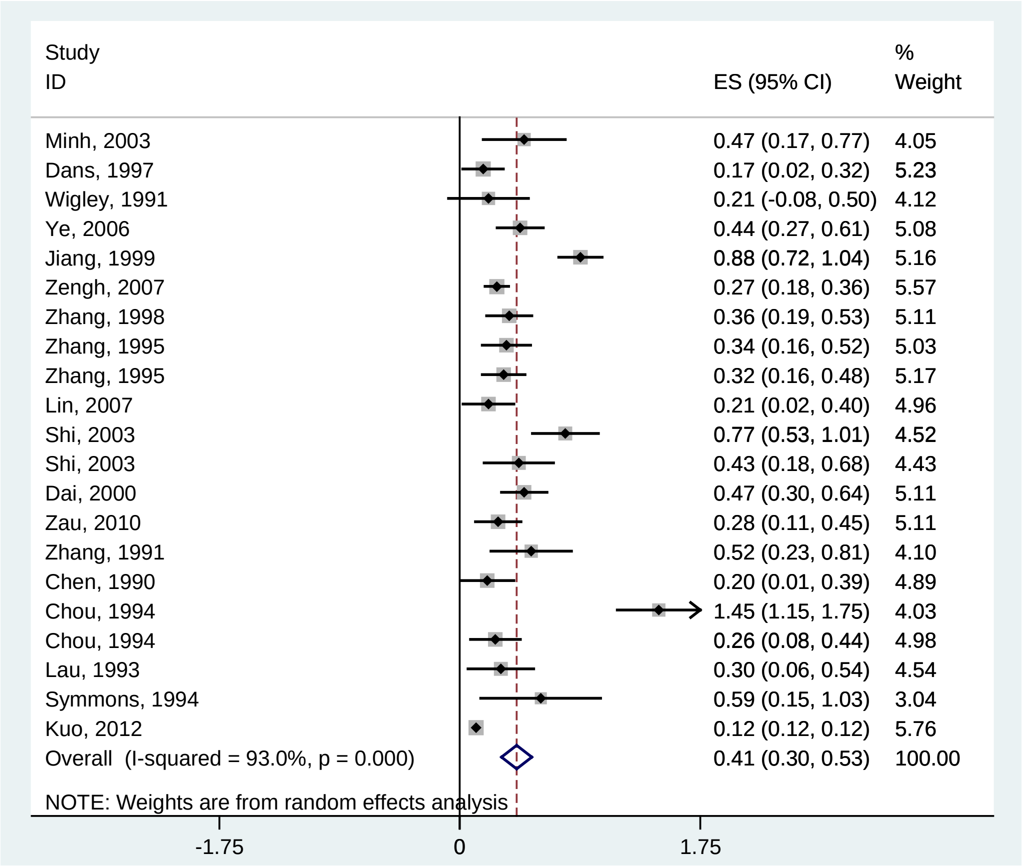

5. Association between mean age of the sample and RA prevalence

The global RA prevalence and the mean age are not associated based on linear correlation coefficients (Pearson, Kendall's tau, and Spearman) and also based on the generalized dependence measure mutual information (p=0.0599; p>0.05).

Coefficients of correlation

		prevalence
mean age	Pearson Correlation	.381
	Sig. (2-tailed)	.060
	N	25
	Kendall's tau	0.065
	Sig. (2-tailed)	0.655
	N	25
	Spearman's rho	0.094
	Sig. (2-tailed)	0.654
	N	25

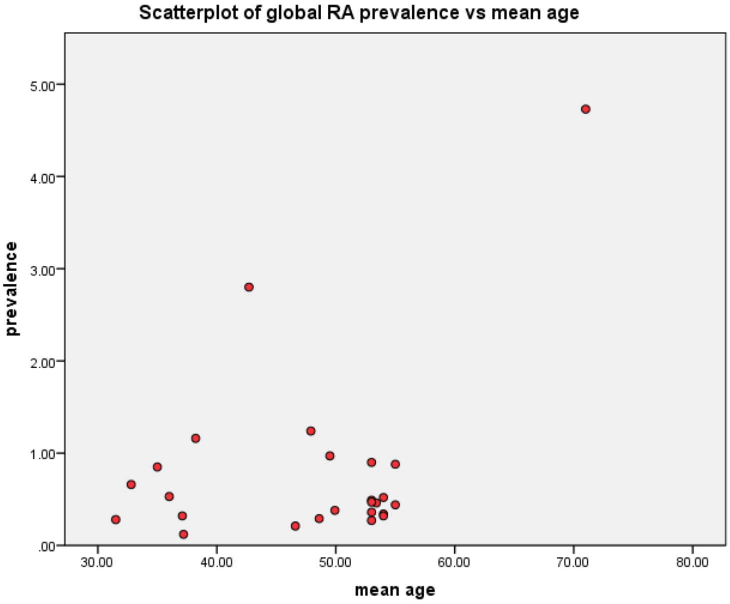

The optimal regression model for the two variables has been estimated after transforming the RA prevalence by taking its square root (sqrt). The variables sqrt (prevalence) and mean age were both normally distributed (p=0.186 and p=0.310, respectively, from the one-sample Kolmogorov Smirnov test).

One-Sample Kolmogorov-Smirnov Test

		mean age	sqrt(prev)
N		25	25
Normal Parameters[a,b]	Mean	47.7776	.8058
	Std. Deviation	9.29083	.39576
Most Extreme Differences	Absolute	.193	.218
	Positive	.178	.218
	Negative	-.193	-.155
Kolmogorov-Smirnov Z		.965	1.089
Asymp. Sig. (2-tailed)		.310	.186

a. Test distribution is Normal.

b. Calculated from data.

The coefficient of determination of the corresponding quadratic model is R^2 = 43.7%, meaning that 43.7% of the variance of the dependent variable, i.e. sqrt (prevalence), is explained by the mean age. The model is given by the equation:

sqrt(prevalence) = -0.001 (mean age)^2 + 0.00002197 (mean age)^3 + 1.668

Model Summary

R	R Square	Adjusted R Square	Std. Error of the Estimate
.661	.437	.386	.310

The independent variable is mean age.

ANOVA

	Sum of Squares	df	Mean Square	F	Sig.
Regression	1.644	2	.822	8.552	.002
Residual	2.115	22	.096		
Total	3.759	24			

The independent variable is mean age.

Coefficients

	Unstandardised Coefficients		Standardised Coefficients	t	Sig.
	B	Std. Error	Beta		
mean age ** 2	-.001	.001	-3.368	-2.935	.008
mean age ** 3	2.197E-005	.000	3.801	3.312	.003
(Constant)	1.668	.424		3.930	.001

6. Association between gender and RA prevalence

Statistics

Male RA prevalence

N	Valid	34
	Missing	0
Mean		.2456
Median		.1500
Std. Deviation		.38030
Minimum		.00
Maximum		2.17

Statistics

Female RA prevalence

N	Valid	33
	Missing	0
Mean		1.0330
Median		.6700
Std. Deviation		1.24794
Minimum		.00
Maximum		7.14

6.1. Paired samples t-test

The paired samples t-test indicates that the male and female RA prevalence differ significantly ($p<0.0001$).

Paired Samples Test

	Paired Differences					t	df	Sig. (2-tailed)
	Mean	Std. Deviation	Std. Error Mean	95% Confidence Interval of the Difference				
				Lower	Upper			
male – female prev.	-.78182	.93556	.16286	-1.11355	-.45008	-4.801	32	.000

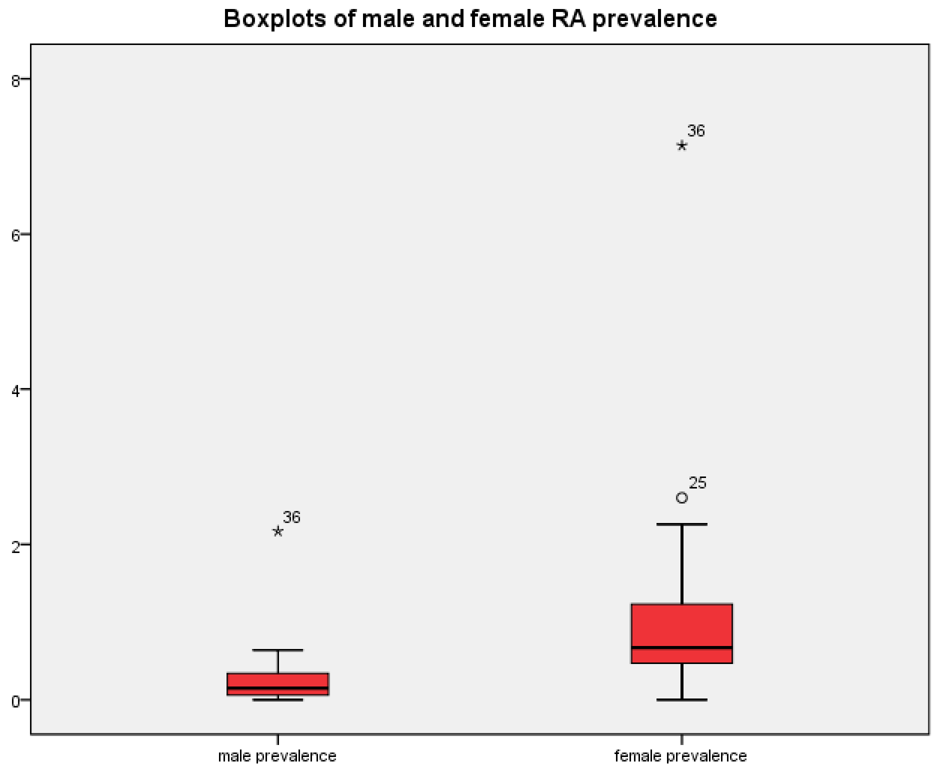

6.2. Meta-analysis of RA prevalence in men

The meta-analysis estimate of the global RA prevalence for men is 0.156 (95% CI: 0.111-0.200) and for women 0.747 (95% CI: 0.598-0.897). Data seem to be moderately heterogeneous for men (I^2 = 49.6%), but substantially heterogeneous for women (I^2 = 83.7%).

6.3. Prevalence in men and women by region

area		Male prevalence	Female prevalence
Southeast Asia	N	4	3
	Mean	.0525	0.65
	Median	.0300	0.55
	Std. Deviation	.07089	0.47791
	Range	.15	0.94
Eastern Mediterranean	N	6	6
	Mean	.2000	0.4433
	Median	.1700	0.43
	Std. Deviation	.09960	0.22888
	Range	.24	0.67
Europe	N	9	9
	Mean	.2267	1.2267
	Median	.1500	1.0400
	Std. Deviation	.24316	0.78102
	Range	.64	2.20
America	N	4	4
	Mean	.7850	2.7675
	Median	.4400	1.6300
	Std. Deviation	.93853	2.95083
	Range	2.08	6.47
Western Pacific	N	9	9
	Mean	.1567	0.6611
	Median	.1500	0.5900
	Std. Deviation	.12329	0.30726
	Range	.40	1.06
Africa	N	2	2
	Mean	.1750	0.7100
	Median	.1750	0.7100
	Std. Deviation	.24749	1.00409
	Range	.35	1.42

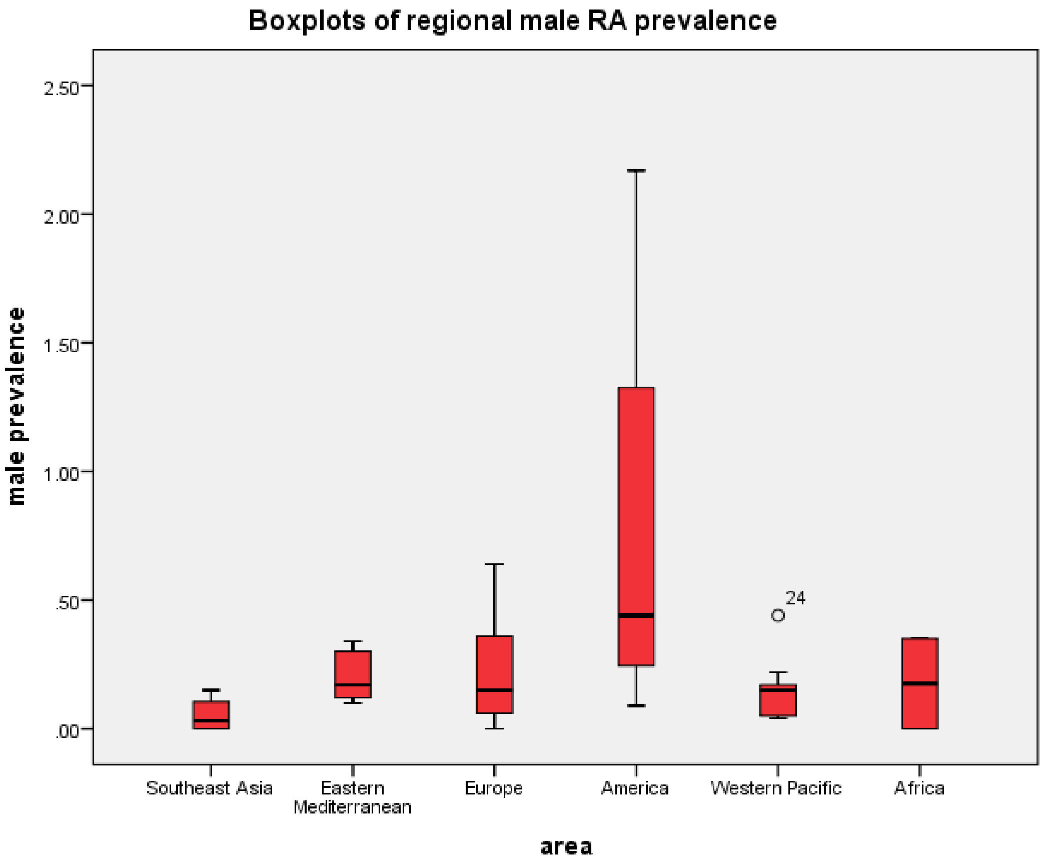
Boxplots of regional male RA prevalence
male prevalence
2.50
2.00
1.50
1.00
.50
.00
24
Southeast Asia
Eastern Mediterranean
Europe
America
Western Pacific
Africa
area

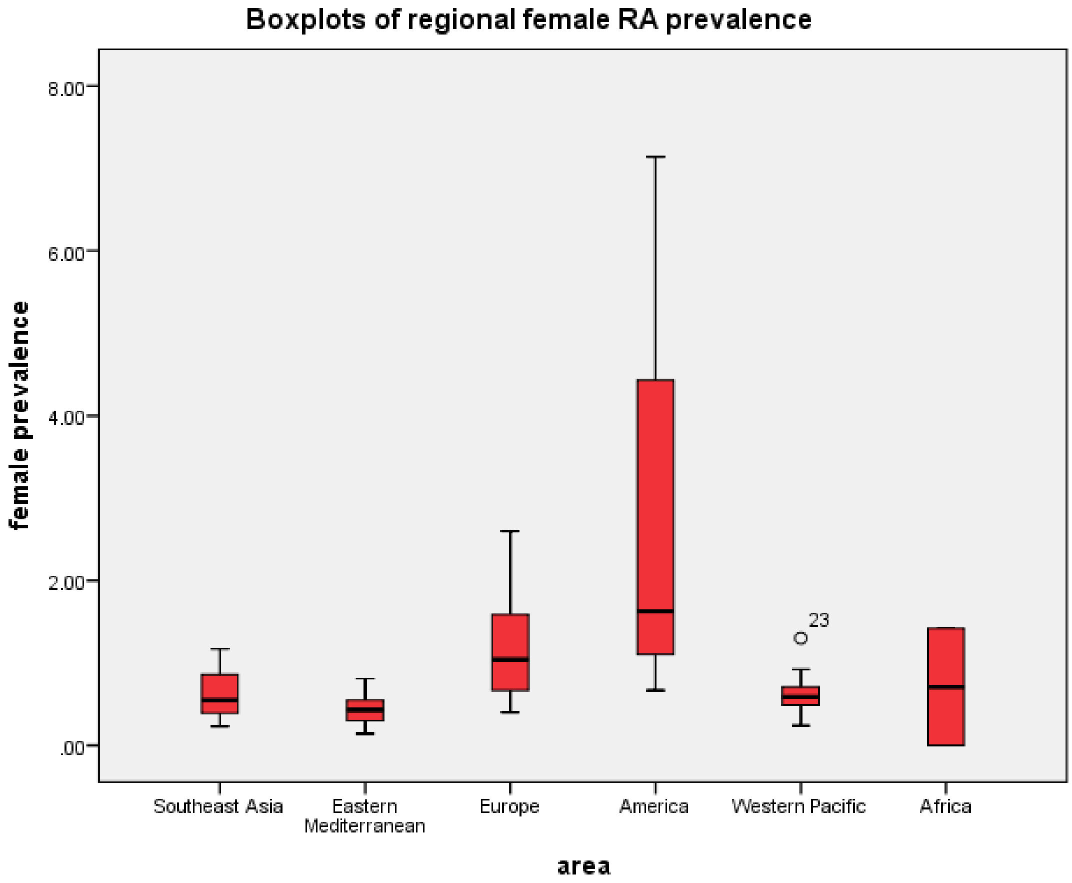
Boxplots of regional female RA prevalence
female prevalence
8.00
6.00
4.00
2.00
.00
23
Southeast Asia
Eastern Mediterranean
Europe
America
Western Pacific
Africa
area

6.4. A summary of meta-analyses (where a sufficient amount of information was available) to determine prevalence by gender and WHO region

	**Regional RA prevalence**		**I^2**	
	**Men**	**Women**	**Men**	**Women**
**Southeast Asia**	-	0.590 (0.105, 1.075)	-	76.8%
**Eastern Mediterranean**	0.134 (0.073, 0.196)	0.396 (0.215, 0577)	0%	64.5%
**Europe**	0.163 (0.070, 0.256)	1.025 (0.661, 1388)	37.5%	81%
**America**	0.494 (-0.090, 1.079)	2.413 (0.776, 4.049)	80.4%	93.6%
**Western Pacific**	0.140 (0.067, 0.214)	0.646 (0.434, 0.858)	67.7%	80.1%
**Africa**	-	-	-	-

7. Global RA prevalence for urban / rural environment

Environment

		Frequency	Percent
Valid	Urban	25	52.1
	Rural	23	47.9
	Total	48	100.0

Descriptive statistics

environment				Statistic
prevalence	urban	Mean		.5700
		95% Confidence Interval for Mean	Lower Bound	.4105
			Upper Bound	.7295
		Median		.4700
		Std. Deviation		.38634
		Minimum		.14
		Maximum		1.60
		Interquartile Range		.39
	rural	Mean		.4874
		95% Confidence Interval for Mean	Lower Bound	.3207
			Upper Bound	.6541
		Median		.3600
		Std. Deviation		.38557
		Minimum		.00
		Maximum		1.53
		Interquartile Range		.51

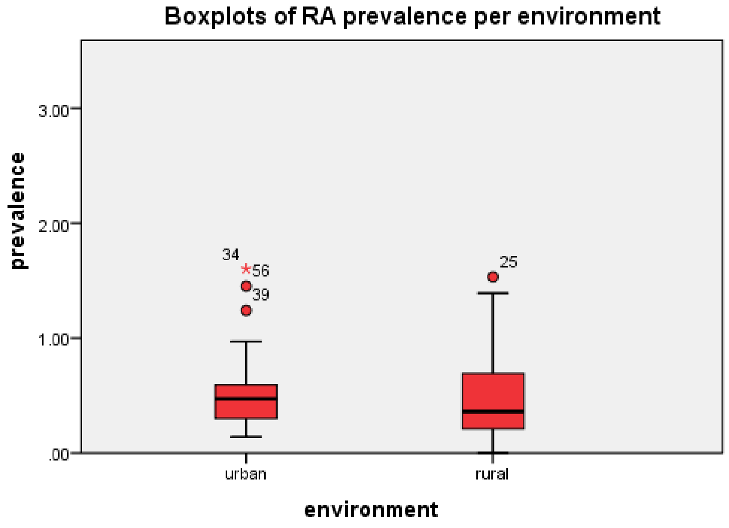

7.1. Mann-Whitney U test

Since the RA prevalence is not normally distributed, we performed the non-parametric Mann-Whitney U test to test the null hypothesis that the two populations are the same. The significance of the test is p=0.353 (p>0.05), indicating that the prevalence in the urban and rural settings do not differ significantly.

Ranks

	Environment	N	Mean Rank	Sum of Ranks
Prevalence	Urban	25	26.30	657.50
	Rural	23	22.54	518.50
	Total	48		

Test Statistics[a]

	prevalence
Mann-Whitney U	242.500
Wilcoxon W	518.500
Z	-.929
Asymp. Sig. (2-tailed)	.353

a. Grouping Variable: environment

7.2. Regional RA prevalence for urban / rural environment

	environment	urban	rural
	area	N	N
prevalence	Southeast Asia	3	4
	Eastern Mediterranean	3	3
	Europe	4	5
	America	6	2
	Western Pacific	9	8
	Africa	0	1

Descriptive statistics

		environment	urban	rural
	area		Statistic	Statistic
prevalence	Southeast Asia	Mean	.38	0.43
		Median	.29	0.42
		Std. Deviation	.16	0.32
		Range	.28	0.63
	Eastern Mediterranean	Mean	.32	0.40
		Median	.35	0.29
		Std. Deviation	.16	0.29
		Range	.32	0.54
	Europe	Mean	.69	0.94
		Median	.71	0.72
		Std. Deviation	.30	0.49
		Range	.59	1.04
	America	Mean	.79	0.48
		Median	.51	0.48
		Std. Deviation	.51	0.53
		Range	1.20	0.75
	Western Pacific	Mean	.52	0.33
		Median	.47	0.33
		Std. Deviation	.40	0.11
		Range	1.28	0.31

Note: For Africa, there are no observations of RA prevalence for urban environment. In the case of rural environment there was only one observation.

7.3. Mean RA prevalence by WHO region and urban/rural environment.

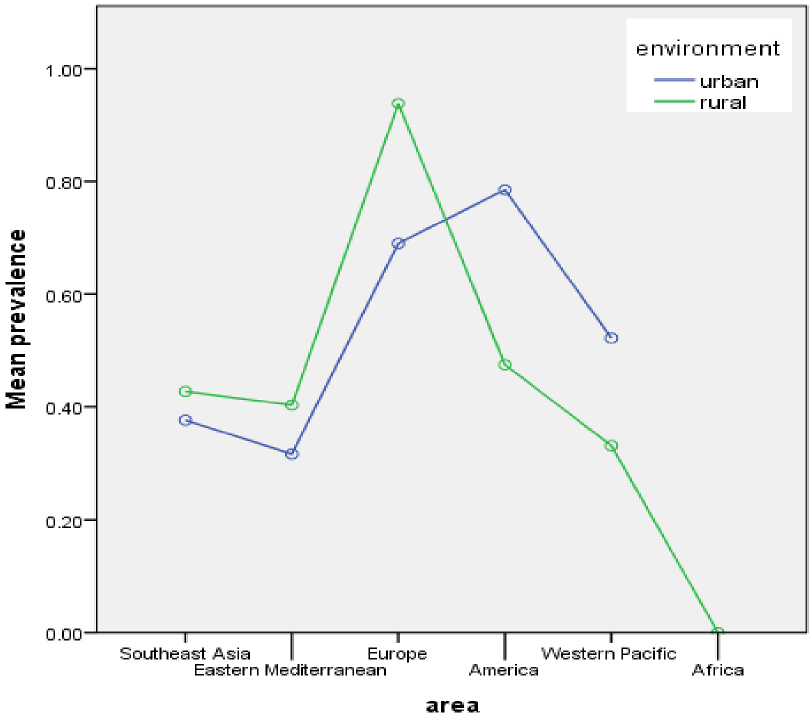

7.4. Median RA prevalence by WHO region and urban/rural environment.

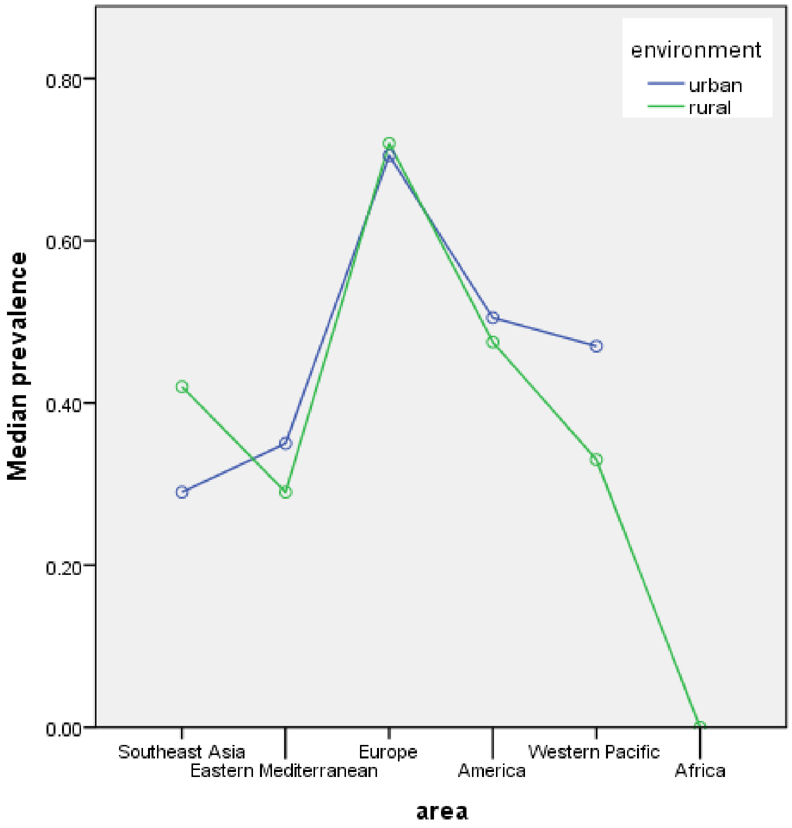

8. The full information extracted from the identified studies and used in a systematic analyses

AUTHOR	COUNTRY/ REGION	URBAN/ RURAL	SAMPLE SIZE	DEFINI-TION	CRUDE PREVA-LENCE	MEAN AGE	MALE PREVALEN-CE RATE	FEMALE PREVALEN-CE RATE	MALE/ FEMALE RATIO
**SOUTHEAST ASIA**									
Chaiamnuay P et al (1998)	Thailand villages of Nakornayok Province	Rural	2463 (2455 respondents 99.7%)	ACR	n = 3; (0.12%)	37.2	1167; n = 0; (0%)	1288; n = 3; (0.23%)	
Darmawan J et al (1993)	Indonesia, central Java	Rural	4683 (95.2% response rate)	ARA	n = 8; (0.18%)		2184	2499	1:3
		Urban	1071 (97.1% response rate)		n = 3; (0.29%)		481	590	1:2
Malaviya AN et al (1993)	India, 5 villages in Haryana	Rural	44551; respondents = 39826	ARA 1987	n = 299; (0.75%)	Maximum prevalence in age group of 25-29	29 cases	270 cases	1:9.3
Chopra A et a l(1988)	India	Urban	110 adults with polyarthritis 16000	ARA	n= 89; (0.56%)	90% aged 25-45	0.06%		

Chopra A et al (2012)	India 12 sites Jammu, Delhi, Guwahati, Bikaner, Manipur, Kolkata, Pune, Hyderabad, Ralegaon Sidhi, Calicut, Chennai, Trivandrum, Bhigwan	Urban & Rural	56,541	ACR	n =198; (0.38%)				
Haq SA et al (2005)	Bangladesh, Bhargaon union	Rural	2635; 98.7% response rate (n=2586)	ACR	0.66%	32.8+/- 15.6	1295; n = 2; (0.15%)	1273 n = 15; (1.17%)	1:7.63
	Bangladesh, Mohammadpur	Urban	2579; respondents =2509		0.28%	31.5. +/- 13.5	1247; n = 0; (0%)	1262; n = 7; (0.55%)	
**EASTERN MEDITTEREAN**									
Chaaya M et al (2012)	Lebanon, 26 districts		3530; 83% response rate	ACR	n = 34; (1.16%)	38.22 +/- 16.91	1804; (0.2%)	1726; (0.3%)	

Davatchi F et al (2009)	Iran, Tehrain (22 districts)	Urban	10,291	ARA	n = 35; (0.33%)		4878; n = 5; (0.09%)	5413; n= 30; (0.58%)	
Davatchi F eta al (2009)	Iran, 5 villages in Tuyserkan County	Rural	1565 86.7% response rate.		0.19%				
Farooqi A & Gibson T (1998)	Northern Pakistan village	Rural	683	ARA	n = 5; (0.73%)		1005, n=3; (0.29%)	986, n = 8; (0.81%)	01:02.7
		Urban	1314		n = 6; (0.46%)				
Hameed K , Gibson T et al (1995)	Pakistan, Karachi (Gulshan & Orangi)	Urban	4232	ARA 1958 criteria & 1987	n = 6; (0.14%)		2156, n =3; (0.14%)	2076 ,n = 3; (0.14%)	01:01
Dahamsheh Z et al (2011)	Jordan, South Jordan		2220	ARA	n= 8; 0.36%		1,185; n = 4; (0.34%)	1035; n = 4; (0.39%)	1:1.14
Abdel-Tawab et al (2004)	Egypt, Mokasa village southern Egypt	Rural	5208, respondents = 5120	ARA	n= 15; (0.29%:)	48.6 +/- 17 years	2558; n = 3; (0.12%)	2562; n= 12; (0.47%)	1:4
Forghanizadeh J et al (1995)	Iran, Fasham-Shemiranat		2502	ACR	0.32%				
**EUROPE**									

Cakir N et al (2012)	Turkey, Havsa	Rural & Urban	12461	ARA	n = 40; (0.32%)	37.1+/- 21.1 years	6040; n = 9; (0.15%)	6445; n = 31; (0.48%)	1.3.2
Capkin E et al (2010)	Turkey	Urban	6103	ACR	n =59; (0.97%)	49.5 +/- 13.8 years	3080; n =11; (0.35%)	3023; n = 48; (1.59%)	
Damjanovic V et al (2009)	Herzegovina	Urban & Rural	28715 from Mostar, 10363 from Siroki Brijeg, 4933 from Stolac and 6676 Grude	ACR	0.46%				01:06
Adomaviciute D et al (2008)	Lithuania, Vilnius & Kaunas	Urban	2101 in Vilnius & 2143 in Kaunas; 4244	ACR	n = 39; (0.92%)		1083; n = 0; (0%)	3161; n = 39; (1.23%)	
Kacar C et al (2005)	Turkey, Antalya	Urban	3173	1987	n =12; (0.38%)	49.92 +/- 11.56 years	1529; n =1; (0.06%)	1644, n =11; (0.67%)	1:10.29
Akar et al (2004)	Turkey, Izmir (Balcova & Narlidere districts)	Urban	2887; 98.2% response rate (2835)	ACR	n =14 (0.49%)	53.0 (+/- 12.4) years	1284; n= 2; (0.15%)	1551; n= 12; (0.77%)	1:4.81

Boyer GS et al (1998)	Siberian Eskimo	Rural	320	1987	1.53%		134; n= 0; (0%)	192; n =5; (2.60%)	
	Chuckchi of Chukotka Pennisula in Russia		405		0.49%		n= 156 1 case 0.64 %	n= 249 1 case 0.40%	1:4.06
	North Slope Borough Inupiat		1651		1.39%		855 5; cases 0.58%	n = 796 18 cases 2.26	1:1.39
	Bristold Bay Yupik Eskimo of Alaska		2135		0.56%		n = 1076; 1 case 0.09%	n= 1059, 11 cases 1.04%	1:11.20
Erdes Sh et al (1999)	Siberia, eastern Chutkotka	Rural	974		n =7; (0.71%)				
**AMERICAS**									
Rodrigeuz-Amado J et lt (2011)	Mexico; Nuevo Leon	Urban	4102	ACR	0.40%	43.6	2074	2639	
		Rural	611		0.10%				
Alvarez-Nemegyei J et al (2011)	Mexico, Yucatan	Urban	1787	ACR	n = 110 (2.8%)	42.7 +/- 17.1	1493	2422	
		Rural	2128						
Senna ER et al (2004)	Brazil; Montes Claros	Urban	3038	ACR	n =14; (0.46%)	53.4 (SD 13.9)	1109; n =1 (0.09%)	1929, n =13 (0.67%)	1:.75
Pelaez-Ballestas I et al (2011)	Mexico: 5 regions	Urban	19,213		1.60%			11602	

Scublinsky D et al (2010)	Argentina, Buenos Aires province	Urban	3742	ARA	0.48%		0.40%	1.54%	
Alvarez Nemegyei J (2005)	Mexico, Southern part (Yucatan)	Urban & Rural	761	ACR	n =36 (4.73%)	71 (range 46-91)	369; n =8; (2.17%)	392; n =28; (7.14%)	1:3.23
Obregon Ponce et al 2012	San Juan Sacatepequez county	Rural	2000	ACR	0.85%	35			
	Guatemala city	Urban	2000		0.53%	36			
Reyes-Llerena et al (2009)	Cuba, Havana City	Urban	3155	ACR	1.24%	47.9 (SD 18.1)	1238, n =6; (0.4%)	1917; n = 33; (1.7%)	
**WESTERN PACIFIC**									
Minh Hoa TT et al (2003)	Vietnam	Urban	2119; 94.4% response rate	ACR	0.47%				
Dans LF et al (1997)	Phillippines, Manila	Urban	3065; response rate of 98%; (3006)	ACR	0.17%				
Wigley R (1991)	Phillippines	Rural	950	ACR	n = 2; (0.21%)				

Ye ZZ et al (2006)	China, Guangdong	Urban & Rural	6684; respondents= 5922	ARA	n =26; 0.44%	55	2659	3262	
Jiang BF et al (1999)	China, Shandong		17044; respondents =13386		n =118; 0.88%	55	6548; n = 29; (0.44%)	6838; n= 89; (1.30%)	1:2.95
Zengh XG et al (2007)	China, Guangxi	Urban & Rural	14233	ARA	n = 39; (0.27%)	53	7497; n= 10; (0.13%)	6736 ;n= 29; (0.43%)	1:3.08
Zhang HK et al (1998)	China, Shangdong	Rural	5820; respondents = 5055	ARA	n= 18; (0.36%)	53	2695; n= 4; (0.15%)	2360; n =14; (0.59%)	1:3.93
Zhang NZ et al (1995)	China, Bejing	Rural	4192; (18 & older)	ARA	n =16; (0.34%)	54	2090; n =1; (0.05%)	2102; n=15; (0.71%)	1:14.2
	China, Guangdong		5057; (18 & older)		n=16; (0.32%)		2384; n=1; (0.04%)	2673; n=15; (0.56%)	1:14
Lin L (2007)	China, Guangodong	Rural	2350	ACR	n=5; (0.21%)	46.6 +/- 15.9	1144; n =2; (0.17%)	1266; n=3; (0.25%)	1:1.47
Shi F et al (2003)	China, Shanghai	Urban	5040; (15 & older)	ARA	39; (0. 77 %)				
		Rural	2558; (15 & older)		11; (0.43%)	#			
Dai SM et al (2000)	China; Shanghai	Urban	6584	ARA	31; (0.47%)	53	3190; n= 7; (0.22%)	3394; n = 24; (0.71%)	1:3.22
Zau JM et al (2010)	China, Shanxi	Urban	3915	ARA	n=11; 0.28%		1858; n=1; (0.05%)	2057; n =10; (0.49%)	1:9.8

Zhang FS et al (1991)	China, Heilongjiang	Rural	2311; (18 & older)	ARA	n=12; (0.52%)	54	1224; n = 2, (0.16%)	1087; n =10; (0.92%)	1:5.75
Chen SL et al (1990)	Shanghai; China	Urban	2020; (15 & older)	ARA	0.20%				1: 1.20
Chou CT et al (1994)	Tiawan	Urban	6000; (20 & older)	ARA	1.45%				1:0.93
		Rural	2998; (20 & older)	ARA	0.26%				1:1.21
Lau E et al (1993)	Hongkong	Urban	2000; (17 & older)	ARA	0.30%				
Symmons DPM (1994)	Hong Kong	Urban	1180	ACR	0.59%				
Kuo CF et al (2012)	Taiwan	Urban & Rural	nationwide;18511000 above 15	ACR	0.12%				
**AFRICA**									
Silman et al (1993)	Nigeria	Rural	127	ACR	0%		0	0	
Malemba et al (2012)	Democratic Republic of Congo		3193 (18 & older)	ACR	0.90%	53	1432; n = 5; 0.35%	1761; n = 25; 1.42%	
Dowman et al (2012)*	African continent	Urban & Rural		ARA & Rome	0.42%				

* This study supersedes all other studies from the African Region
